# Supplementary material for: Cultural evolution by capital accumulation
Source: Evol Hum Sci. 2020 May 7;2:e18. doi: 10.1017/ehs.2020.19 (PMC10427485; doi:10.1017/ehs.2020.19)
Supplement: Supplementary file 1 [file S2513843X20000195sup001.pdf]

Supporting Information for “Cultural evolution by capital accumulation”

Jean-Baptiste André[a, ✉] and Nicolas Baumard[a,]

[a]Institut Jean Nicod, Département d'études cognitives, ENS, EHESS, PSL Research University, CNRS, Paris France

DRAFT

A. Evolutionarily stable schedule.

A.1. Model 1. In model 1, the production function is merely a target that individuals seek to maximize when they invest in different compartments of their capital, but this model deliberately neglects the fact that this function in turn determines the amount of resources that can be invested per unit of time in different forms of capital. It is assumed that, whatever the amount of capital owned by an individual, she produces the same constant amount of resource per unit of time (without loss of generality, this constant is considered equal to 1). This is a deliberately false approximation but it serves as a point of comparison with the next model.

In both models, we assume that individuals follow a bang-bang life history strategy, investing in their growth for a duration L and then in reproduction for the remaining of their life –the duration of which needs not be specified in the model. In principle the time L that individuals devote to growth, before they start to reproduce, should itself be a decision of individuals, evolving by natural selection. However, in the sake of simplicity, we neglect this aspect of resource allocation in both models, and assume that individuals have an exogenously fixed time, L, available to grow. In this case, the evolutionarily stable strategy simply consists in aiming to maximize one's productivity at age L, as this fully determines one's total reproductive success. As long as the production function is concave (which is the case here since we have assumed $\alpha \leq 1$ and $\beta \leq 1$), standard results of optimal control theory (see Iwasa and Roughgarden, 1984, Perrin, 1992, Perrin et al., 1993) tell us that the optimal allocation strategy for individuals to do so consists in investing at all times in the activity (growth, learning, or innovation) that has the greatest marginal impact on productivity (see Iwasa and Roughgarden, 1984, Perrin, 1992, Perrin et al., 1993). From the production function given by the equation 1 of main text, the marginal production of x and y, respectively, are given by

$$P_x(x,y) \equiv \frac{\partial P(x,y)}{\partial x} = \alpha x^{\alpha-1} y^{\beta}$$
$$P_y(x,y) \equiv \frac{\partial P(x,y)}{\partial y} = \beta x^{\alpha} y^{\beta-1}$$

So the marginal return of investing a unit of time in growth is $\gamma P_x(x,y)$, the marginal return of investing a unit of time in learning is $\lambda P_y(x,y)$ and the marginal return of investing a unit of time in innovation is $\eta P_y(x,y)$ with $\eta < \lambda$.

We consider a population of N individuals developing in parallel in an environment in which an amount z of knowledge is already available from the previous generation.

A.1.1. Phase 1: Learning and growth. As long as individuals have not learned all the cultural information already available (that is, as long as $y < z_0$) then the return on innovation is always lower than the return on social learning, and individuals thus only arbitrate between (i) growth and (ii) social learning. During this phase, if $\gamma P_x(x,y) > \lambda P_y(x,y)$ then individuals prefer to invest all their resources in growth, while if $\gamma P_x(x,y) < \lambda P_y(x,y)$ then they prefer to invest all their resources in social learning. Natural selection therefore favours a strategy that consists of investing a fraction of the resources in growth and a fraction in learning in order to maintain at all times $\gamma P_x(x,y) = \lambda P_y(x,y)$, which implies maintaining a ratio

$$\frac{y}{x} = \frac{\lambda\beta}{\gamma\alpha} = \frac{\lambda}{\gamma}\kappa$$

where $\kappa \equiv \beta/\alpha$. To maintain this ratio, individuals invest a fraction of their resources l in social learning and a fraction $1-l$ in growth, with

$$l = \frac{\beta}{\alpha+\beta} = \frac{\kappa}{1+\kappa}$$

The growth rate of x during this phase is therefore $\gamma(1-l)$ while the growth rate of y is λl. This phase lasts until all available knowledge has been learned, so until $y = z$, which takes a time

$$\tau_1(z) = \frac{z}{\lambda}\frac{\kappa+1}{\kappa} \tag{1}$$

At the end of this phase, we have $y = z$ and $x = x_1 = \frac{\gamma}{\lambda\kappa}z$.

A.1.2. Phase 2: Pure growth. Once $y = z$, individuals cannot improve y through social learning. They only have the choice between innovating or growing. However, the efficiency of innovation is lower than the efficiency of social learning (i.e. $\eta < \lambda$), which implies $\eta P_y(x,y) < \lambda P_y(x,y)$. At the end of the first phase we had equal returns $\lambda P_y(x_1,z) = \gamma P_x(x_1,z)$. This implies $\eta P_y(x_1,z) < \gamma P_x(x_1,z)$. That is, at the end of Phase 1, investing in growth is necessarily more profitable than innovating. So individuals do not start innovating right away. They first experience a phase during which they only invest in the growth of x. During this phase, the return of growth $\gamma P_x(x,y)$ decreases. Hence, this phase ceases when this return meets that of innovation, that is when $\gamma P_x(x,y) = \eta P_y(x,y)$, which implies $x = x_2 = \frac{\gamma}{\eta\kappa}z$, at which point individuals can start innovating (phase 3). This second catch-up

phase therefore lasts for a duration

$$\tau_2(z) = \frac{\lambda - \eta}{\lambda\eta\kappa} z \tag{2}$$

Hence, catching-up takes a total time $\tau(z) \equiv \tau_1(z) + \tau_2(z)$, which gives

$$\tau(z) = \frac{\lambda + \eta\kappa}{\lambda\eta\kappa} z \tag{3}$$

This allows us to define the average growth rate of individuals during the catch-up phase

$$r_c \equiv \frac{z}{\tau(z)} = \frac{\lambda\eta\kappa}{\lambda + \eta\kappa} \tag{4}$$

A.1.3. Phase 3: Innovation, learning and growth, at the frontier. At the end of phase 2, the marginal return on investment in growth is exactly equal to the return on investment in innovation $\gamma P_x(x,y) = \eta P_y(x,y)$. Since social learning is always more efficient than innovating ($\lambda > \eta$) this means that social learning is still the most profitable activity. So, as soon as an individual in the population produces an innovation, then all the other individuals prefer to invest in learning this innovation rather than in any other activity. In other words, during this third phase, individuals do not only invest in innovation and growth, they always keep a fraction (sometimes very large in fact) of their resources to invest in the social learning of *all* the innovations produced by others. The difficulty then consists in calculating the rate at which a population of N individuals produces new knowledge. To do this, let us assume that each individual invests a fraction of his resources d in innovation, a fraction l in social learning, and a fraction $1 - l - d$ in the growth of his personal capital x. Two conditions must be met by d and l.

1. Individuals must learn all innovations produced by others, which implies that l and d must follow the condition $\lambda l = \eta d(N-1)$.

2. Individuals must maintain the respective returns of innovation and growth equal, i.e. $\eta P_y(x,y) = \gamma P_x(x,y)$. This implies that they maintain the ratio $\frac{y}{x} = \frac{\eta}{\gamma}\kappa$. Therefore, l and d must also follow the condition $\frac{\lambda l + \eta d}{\gamma(1-d-l)} = \frac{\eta}{\gamma}\kappa$.

These two conditions can be solved. This gives us $l = \frac{\eta\kappa(N-1)}{\eta\kappa(N-1)+\lambda(\kappa+N)}$ and $d = \frac{\kappa\lambda}{\eta\kappa(N-1)+\lambda(\kappa+N)}$. The speed of cultural evolution during this third phase is $r_i \equiv \lambda l + \eta d = \eta d N$, which therefore gives

$$r_i = \frac{\lambda\eta\kappa N}{\lambda(N+\kappa) + \eta\kappa(N-1)} \tag{5}$$

If a population contains an amount of knowledge z_t in generation t, in the next generation it contains an amount $z_{t+1} = z_t + r_i\left(L - \frac{z_t}{r_c}\right) = r_i L + \frac{r_c - r_i}{r_c} z_t$. Considering the initial condition $z_0 = 0$, this recurrence equation leads to the explicit dynamics of z_t over successive generations:

$$z_t = L r_c \left[1 - \left(\frac{r_c - r_i}{r_c}\right)^t\right] \tag{6}$$

A.2. Model 2. In model 2, it is explicitly considered that the production function determines the amount of resources available per unit of time, that individuals can then allocate to their different activities. Here as well, we assume that individuals have an exogenously fixed time, L, available to grow such that the evolutionarily stable strategy simply consists in aiming to maximize one's productivity at age L. Here as well, as long as the production function is concave, the optimal allocation strategy for individuals is to make, at each instant, the investment(s) with the strongest marginal effect on productivity. This simple principle allows us to simulate the development of individuals.

The problem is that, in model 2, the growth rate of individuals is not constant (unlike model 1) but given by the production function and the development of individuals is therefore not analytically solvable. We therefore simulate numerically step by step this development in a social environment in which they initially have an amount z of available knowledge from the previous generation. These simulations are performed by choosing a time step δ, the smallest possible (ideally infinitely small). At each time step we numerically calculate the marginal return, $P_x(x,y)$ and $P_y(x,y)$, of each type of capital and we calculate the growth of individuals during this time step as follows:

(1) When $y < z$ (that is, individuals still have things to learn socially). (i) If $\lambda P_y(x,y) < \gamma P_x(x,y)$ individuals invest all their production in growth and therefore x increases by $\gamma P(x,y)\delta$ while y remains constant. (ii) If $\lambda P_y(x,y) > \gamma P_x(x,y)$ individuals invest all their production in social learning and therefore y increases by $\lambda P(x,y)\delta$ while x remains constant.

(2) When $y \geq z$ (that is, individuals no longer have anything to learn socially from the previous generation). (i) If $\eta P_y(x,y) < \gamma P_x(x,y)$ individuals invest all their production in growth and therefore x increases by $\gamma P(x,y)\delta$ while y remains constant. (ii) If $\eta P_y(x,y) > \gamma P_x(x,y)$ individuals invest all their production in both (i) innovating and (ii) learning the innovations made by others. In this case, y increases by $\frac{\eta\lambda N}{\lambda+\eta(N-1)} P(x,y)\delta$ while x remains constant.

With these simulations, by considering a population of N individuals with a quantity of knowledge z_t available in generation t, we can simulate the development of individuals and thereby obtain the quantity of knowledge that they reach at the end of their development, which gives us z_{t+1}, the quantity of knowledge available for the next generation. Eventually, performing the same simulations for different values of z_t allows us to plot z_{t+1} in function of z_t (Figs. 4 and 5 of main text).

In the particular case where the relative importance of x and y depends on the capital of individuals (section "Investments in knowledge increase along the pyramid of needs" of main text), we assume that the two exponents, α and β , of the production function (Eq. 1 of main text) depend on non-heritable capital, via two functions $\alpha(x) = (1 - c(x))\zeta$ and $\beta(x) = c(x)\zeta$, with $\zeta \in [0.1]$ and $c(\cdot)$ an increasing monotonic mapping from $[0. +\infty]$ to $[0.1]$. The model is then exactly the same except that the marginal productivities of x

DRAFT

and y are given by

$$P_x(x,y) = \delta x^{\zeta(1-c(x))-1} y^{\zeta c(x)} \left(-xc'(x)\ln\left(\frac{x}{y}\right) - c(x) + 1\right)$$
$$P_y(x,y) = \zeta c(x) x^{\zeta(1-c(x))} y^{\zeta c(x)-1}$$

B. Division of labour. Here we consider a model in which individuals share the work of learning and producing cultural goods. We assume that they do so in a cooperative manner by seeking the most efficient division of labour possible. When the size of the population and the discrete nature of individuals does not constrain the division of labour, then the result under this assumption is identical to the long-term equilibrium of a competitive market (see Mas-Colell et al., 1995, chapter 10).

We consider a population of N individuals and we consider to simplify a good for which the demand is constant. The good has a benefit b and each of the N individuals in the population needs exactly one and only one token of this good. Among the N individuals in the population, n are producers of the good (and also consumers like everyone else). The ratio N/n therefore measures the extent of the division of labour. The higher it is, the more extensive is division of labour.

The cost of producing the goods is two fold. First, being a producer entails a "fixed cost", ϕ, that is independent of the amount of goods actually produced, and represents the cost of acquiring the knowledge and skills to make the good, and more generally all the fixed costs necessary to become a producer. Second, being a producer entails a "production cost", $\pi(q)$, that depends on the number q of goods produced. Typically, the production cost function should be convex, at least at some point, such that it becomes too costly to produce more goods (see Mas-Colell et al., 1995, chapter 10). Here, we consider a standard production cost

$$\pi(q) = uq^a \tag{7}$$

where u is the cost of producing one unit of the good and $a > 1$ measures the convexity of the production cost.

Let us first assume that there are exactly n producers of the good in the population. Every producer will have to produce exactly N/n goods that they sell at a price p. In this case, non-producing consumers earn a payoff $b-p$. And producers earn a payoff $g(p,n)$ given by

$$g(p,n) = p\frac{N}{n} - \left(\phi + \pi\left(\frac{N}{n}\right)\right) + b - p \tag{8}$$

We assume that the number of producers, n, and the price of the good p are (i) in equilibrium and (ii) efficient. They must therefore comply with two conditions.

(1) Equilibrium condition: The price of the good is such that producers and consumers earn the same payoff. Otherwise some consumers would prefer becoming producers, or vice versa (Mas-Colell et al., 1995, chapter 10.F), and n would therefore not be in equilibrium. This implies the condition $p\frac{N}{n} = \phi + \pi\left(\frac{N}{n}\right)$, which gives us the equilibrium price $\hat{p}(n)$ at which the good is sold when there are n producers:

$$\hat{p}(n) = \frac{n}{N}\left(\phi + \pi\left(\frac{N}{n}\right)\right) \tag{9}$$

When the good is sold at the equilibrium price $\hat{p}(n)$, individuals share the cost of producing the goods in such a way that everyone earns the same payoff given by $b - \hat{p}(n)$.

(2) Efficiency condition. The number of producers n should be such that the average payoff of individuals is maximized when the good is sold at the equilibrium price $\hat{p}(n)$. In combination with the condition 9 above, this implies that the $\hat{p}(n)$ is minimized. So this gives us

$$\hat{n} = \arg\min_n \left(\frac{n}{N}\left(\phi + \pi\left(\frac{N}{n}\right)\right)\right) \tag{10}$$

From these two conditions, and with the production cost given by equation 7, we obtain the optimal number of producers $\hat{n}$ given by

$$\hat{n} = \frac{N}{\hat{s}} \text{ if } \hat{s} \in [1,N] \tag{11}$$
$$\hat{n} = N \text{ if } \hat{s} \leq 1 \tag{12}$$
$$\hat{n} = 1 \text{ if } \hat{s} \geq N \tag{13}$$

with

$$\hat{s} = \left(\frac{\phi}{(a-1)u}\right)^{1/a} \tag{14}$$

In other words, $\hat{s}$ corresponds to the optimal division of labour in the absence of constraints (i.e. when the size of the population and the discrete nature of individuals are not limiting). $\hat{s}$ increases with the fixed cost ϕ and decreases with a and u which is expected since production techniques are less efficient. The actual division of labour is, however, constrained by (i) population size (at most all individuals will be producers) and above all by (ii) the discrete nature of the individuals (at least there must be 1 producer).

At the optimum division of labour, the price of the good is thus given by

$$\hat{p} = a \cdot u \left(\frac{l}{(a-1)u}\right)^{\frac{a-1}{a}} \text{ if } \hat{s} \in [1,N] \tag{15}$$
$$\hat{p} = u + \phi \text{ if } \hat{s} \leq 1 \tag{16}$$
$$\hat{p} = \frac{uN^a + \phi}{N} \text{ if } \hat{s} \geq N \tag{17}$$

This prize is then integrated into the cultural evolution model as follows.

First, the fixed cost of becoming a producer, ϕ, represents the amount of cultural information needed to become a producer. We assume that this cost is simply given by $\phi = z$. Second, we assume that the greater the amount of information, z, available in the previous generation, the more efficient are the techniques for producing goods. This is modelled by assuming that the convexity of the production cost function (eq. 7) is a decreasing function of the technical

DRAFT

complexity z, equal to a_{high} when $z = 0$, and asymptotically reaching a_{low} when z tends toward infinity:

$$a(z) = a_{\text{high}} - z(a_{\text{high}} - a_{\text{low}}) \frac{\sigma}{a_{\text{high}} - a_{\text{low}} + z\sigma} \quad \textbf{(18)}$$

During the catch-up phase, when individuals still have less knowledge than the amount of knowledge available in the previous generation ($y < z$), the effective learning rate is given by $\lambda(z) = \lambda_{\min} \cdot \max(1, \frac{z}{p(z)})$. At worst, when the price of cultural goods is very high, the learning rate is $\lambda_{\min}$. When z increases, the efficiency of production techniques improves so that the division of labour is greater (the number of producers decreases), the price of goods decreases ($p(z) < z$), and thus the effective learning rate increases (i.e. $\lambda(z) > \lambda_{\min}$). When individuals are innovating at the frontier, however, then we assume that their learning rate is constant, and given by $\lambda_{\min}$.

Iwasa, Y. and J. Roughgarden, 1984. Shoot/Root Balance of Plants: Optimal Growth of a System with Many Vegetative Organs. Theoretical Population Biology 105:78–105.

Mas-Colell, A., M. D. Whinston, and J. R. Green, 1995. Microeconomic theory. Oxford University Press, Oxford.

Perrin, N., 1992. Optimal Resource Allocation and the Marginal Value of Organs. The American Naturalist 139:1344–1369.

Perrin, N., R. M. Sibly, and N. K. Nichols, 1993. Optimal growth strategies when mortality and production rates are size-dependent. Evol. Ecol. 7:576–592.

DRAFT
